# Chronic Cerebral Abscess

**Published:** 1895-03-23

**Authors:** 


### CHRONIC CEREBRAL ABSCESS.

By M.B.

Suppuration within the cranial cavity is one of those conditions, the possibility of which must ever be borne in mind. The great tact and nicety of discrimination often required in the diagnosis of these cases is well known. Our knowledge of the signs of cerebral disturbance and our refinements in cerebral localisation have enormously increased in recent years. So much so that in some instances our diagnosis may appear to be absolutely certain. Yet, when the skull is opened on post-mortem examination, the conditions found may be a perfect surprise. In tumour, hæmorrhage, or meningitis, we may be able to come to an accurate conclusion; but, in the case of abscess, it is no such easy matter. More especially is this difficulty of diagnosis present in chronic cerebral abscess. In recent numbers of the *Lancet* we have an admirable illustration of this point. Professor Murri, in his exceedingly interesting and instructive address before the Lombard Medical Association, goes very fully into this matter, and his cases display in a remarkable degree, not only the value of a nice sense of discrimination, but also the necessity, in many cases, of "exploratory incision."

As Professor Murri points out, in spite of the incompleteness of our powers of diagnosis, our treatment of chronic cerebral abscess has made great advance of late years. We can treat a chronic cerebral abscess, but we do not know how to recognise it. Acute cerebral abscess and traumatic purulent meningitis are often easily made out, but the more chronic forms may be exceedingly difficult of discovery. We may have a history of injury to the head, of purulent otitis of heart or lung disease, or of caries of the skull bones; but these will not prove the presence of abscess. On the other hand, causes so slight as almost to exclude such a possibility may be followed by abscess. In other words, chronic cerebral abscess may be present and give absolutely no sign of its existence, and may only be discovered post-mortem; or symptoms pointing to this condition may be present and the patient die from meningitis or neoplasm.

The signs by which we might expect to diagnose this condition are not by any means numerous or trustworthy.

Fever is not always present; indeed, according to Murri, a subnormal temperature is a much more reliable sign than pyrexia, and is much more likely to be overlooked.

Headache, even if present, which is by no means always the case, is not diagnostic of this condition. Optic neuritis may be equally suggestive of tumour or meningitis. Pressure symptoms are but little help beyond localisation, they do not tell us the nature of the disturbing element. In short, it is only by carefully weighing all the signs and symptoms that may present themselves that we can be at all confident as to the presence of chronic cerebral abscess, and even then we cannot by any means be certain as to our diagnosis.

This being the case, we can understand how it is that these cases are now coming more and more under the care of the surgeon. We have reason to believe that there is some disturbance going on within the skull. But what is its nature? The abdomen is frequently explored with impunity, why not the skull? It is gratifying to know that this has now been done in a good many cases, with results that are eminently satisfactory. Chronic cerebral abscess, if left alone, is almost certain sooner or later to prove fatal, and it is a plea for more frequent "experimental craniotomy" that forms the text of Professor Murri's address, and we are pleased to note the high praise accorded to our British surgeons for their bold, but at the same time well thought-out, advance in cerebral surgery, which enables them, by the timely use of the trephine, not merely to clear up the diagnosis of an obscure case, but to rescue in many instances from the jaws of death a class of patients who, without this interference, must inevitably succumb to the disease.

